# Boron Neutron Capture Therapy for High-Grade CNS Tumors: Mechanisms, Carriers, and Clinical Progress: A Narrative Review

**DOI:** 10.3390/ijms27062765

**Published:** 2026-03-18

**Authors:** Tugce Kutuk, Ece Atak, Marshall Harrell, Raju R. Raval, Fatemeh Fekrmandi, Simeng Zhu, Sasha Beyer, Pawan K. Singh, Pierre Giglio, Hamid Mohtashami, Kyle C. Wu, James Bradley Elder, Sean S. Mahase, Raj Singh, Arnab Chakravarti, Joshua D. Palmer

**Affiliations:** 1 Department of Radiation Oncology, The Ohio State University Wexner Medical Center, Columbus, OH 43210, USA; 2 Department of Radiation Oncology, Basaksehir Cam and Sakura City Hospital, Istanbul 34480, Türkiye; 3 Division of Neuro-Oncology, Department of Neurology, The Ohio State University Wexner Medical Center, Columbus, OH 43210, USA; 4 Department of Neurological Surgery, The Ohio State University Wexner Medical Center, Columbus, OH 43210, USA; 5 Department of Radiation Oncology, Penn State Cancer Institute, Hershey, PA 17033, USA; 6 Department of Radiation Oncology, Lynn Cancer Institute, Baptist Health South Florida, Boca Raton, FL 33486, USA

**Keywords:** boron neutron capture therapy (BNCT), glioblastoma, high-grade glioma, meningioma, CNS tumors

## Abstract

Boron neutron capture therapy (BNCT) is a biologically targeted, high–linear energy transfer radiotherapy that selectively delivers cytotoxic α-particles to boron-loaded tumor cells and has re-emerged with the development of hospital-compatible accelerator neutron sources and improved boron carriers. We performed a structured literature review of PubMed, Embase, and the Cochrane Library through October 2025 to summarize the radiobiological rationale, boron delivery strategies, and clinical outcomes of BNCT in glioblastoma (GBM) and other high-grade central nervous system tumors. Eligible clinical and translational studies were screened independently, and data on patient populations, boron agents, neutron source technologies, dosimetry, survival, response, and toxicity were extracted. Contemporary series and phase II trials indicate that BNCT is technically feasible and generally well tolerated, with encouraging survival outcomes in selected newly diagnosed and recurrent GBM, meaningful activity in recurrent high-grade meningiomas, and acceptable safety in limited pediatric cohorts. Current practice relies primarily on second-generation carriers such as boronophenylalanine and sodium borocaptate, while third-generation molecular and nanocarrier platforms remain in preclinical development. Overall, BNCT represents a promising high-LET, pharmacologically targeted modality for heavily pretreated and radioresistant CNS tumors, and ongoing prospective studies are needed to define its comparative effectiveness and optimal integration into patient care.

## 1. Introduction

Glioblastoma (GBM) is the most common primary malignant central nervous system (CNS) tumor in adults. Following maximally safe resection, fractionated external beam radiotherapy with concomitant and adjuvant temozolomide is the current standard of care [1,2]. Subsequently, Tumor Treating Fields (TTFields) emerged as the only modality with randomized phase III evidence demonstrating a survival benefit to the aforementioned first-line setting standard of care, extending median overall survival (OS) from 16.0 to 20.9 months [3]. However, five-year OS remains below 10%, underscoring the need for novel therapeutic strategies [4].

Multiple radiotherapeutic strategies were explored in the newly diagnosed and recurrent setting. Despite exploratory signals with dose-escalated proton therapy in NRG-BN001, randomized comparisons failed to demonstrate an OS advantage over photon-based radiotherapy [5]. Notably, secondary analyses suggested a trend toward improved local control and survival in selected subgroups receiving higher biologically effective doses, although these findings were not statistically significant and should be considered hypothesis-generating. In the re-irradiation setting, pulsed-reduced dose-rate (PRDR) radiotherapy demonstrated feasibility and acceptable toxicity in institutional series, with a median 6-month progression-free survival (PFS) [6]. Carbon-ion radiotherapy, primarily evaluated as a boost following photon or proton therapy [7], and salvage strategies including intraoperative radiotherapy (IORT), Cs-131 brachytherapy, and radioligand-based theranostic, predominantly explored in small, single-arm institutional experiences, limits their potential benefits pending further data [8,9].

Among emerging approaches, boron neutron capture therapy (BNCT) is distinguished by its reliance on pharmacologic tumor targeting [10]. Unlike systemic radioligand therapies, BNCT employs non-radioactive ^10^B-containing compounds that preferentially accumulate within tumor cells, followed by neutron irradiation that triggers the ^10^B(n,α)^7^Li reaction, generating ultra-high LET α-particles and ^7^Li nuclei [11,12]. BNCT offers a non-invasive, high-LET modality selectively targeting infiltrative glioma cells while limiting normal tissue injury. This review provides a synthesis of the radiobiological rationale, delivery strategies, and clinical data analyzing BNCT in GBM and other high-grade CNS malignancies.

A systematic literature search was conducted through October 2025 to identify clinical and preclinical/translational studies reporting outcomes of BNCT in GBM and other high-grade CNS tumors. PubMed, Embase, and the Cochrane Library were searched using combinations of keywords, including “boron neutron capture therapy” OR “BNCT” AND “glioblastoma” OR “high-grade glioma” OR “meningioma” OR “high-grade CNS tumor”. Reference lists of relevant articles and prior reviews were hand-searched to identify additional studies.

## 2. Mechanism of Action

**Figure 1:**
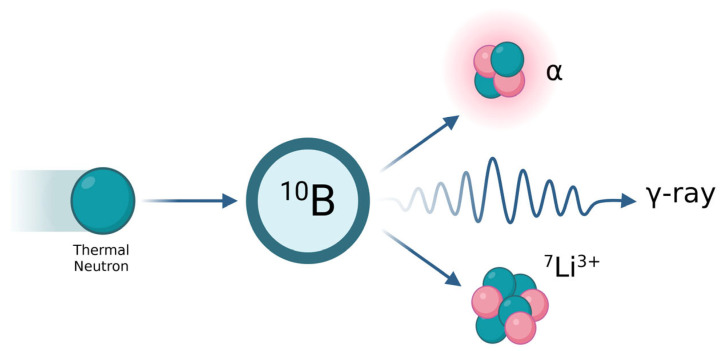
Schematic of the ^10^B(n,α)7Li reaction in BNCT. Thermal neutron capture by ^10^B produces an α-particle and 7Li nucleus, whose ultra-high LET emissions deposit energy over a 5–9 μm range, confining cytotoxicity to boron-loaded tumor cells. Created in BioRender. Atak, E. (2026) https://BioRender.com/xypznkw.

BNCT is a binary radiotherapeutic modality relying on the preferential accumulation of stable ^10^B within tumor cells, followed by irradiation with thermal or epithermal neutrons [13]. Neutron capture initiates the ^10^B(n,α)^7^Li reaction, generating an α-particle (~1.47 MeV) and a recoiling ^7^Li nucleus (~0.84 MeV) with short path lengths of approximately 5–9 μm and a mean LET of ~150 keV/μm, a range smaller than the diameter of a typical human cell, thereby producing highly localized, potent high-LET radiation damage confined to boron-containing cells (Figure 1). Therapeutic efficacy depends on neutron fluence, pharmacokinetics, intracellular uptake, and microdistribution of boron carriers. Achieving favorable tumor-to-blood and tumor-to-normal tissue ratios (typically ≥3:1) is essential for an optimal therapeutic index. Competing neutron-capture reactions from biologically abundant nuclei such as ^12^C, ^1^H, and ^14^N are negligible because their thermal-neutron capture cross sections are orders of magnitude lower than ^10^B [14]. In contrast to proton or carbon-ion therapy, where biological effectiveness is intrinsic to beam properties, BNCT derives its selectivity from biological targeting, creating a unique interdependence between drug delivery and radiation physics. At the cellular level, BNCT induces dense, clustered DNA double-strand breaks, chromosomal aberrations, mitotic catastrophe, and apoptosis, consistent with the ultra-high-LET microdosimetric patterns generated by α-particles and ^7^Li nuclei [15].

## 3. Historical Background

**Figure 2:**
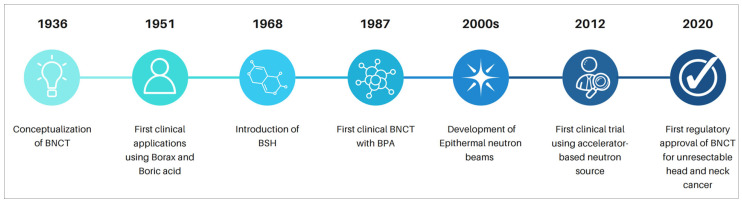
Historical milestones in the development of BNCT. Key advances include Locher’s original conceptualization in 1936, early clinical use of borax and boric acid in the 1950s, introduction of BSH (1968) and BPA (1987), development of epithermal neutron beams in the 2000s, the first clinical trial using an accelerator-based neutron source in 2012, and the first regulatory approval of BNCT for unresectable head and neck cancer in 2020.

The conceptual foundation of BNCT was first proposed by Gordon Locher in 1936, who hypothesized that non-radioactive boron could be used as a neutron-capturing agent to selectively deliver high-LET radiation to tumors (Figure 2) [16,17,18]. The earliest clinical applications in GBM were conducted at Brookhaven National Laboratory and Massachusetts General Hospital in the 1950s, using borax and early inorganic boron salts in conjunction with reactor-based thermal neutron beams [17,18]. While establishing feasibility, these initial trials were limited by the poor tumor selectivity of first-generation boron agents, and suboptimal neutron energy spectra, resulting in inadequate tumor dosing and toxicity. By the late 20th century, these limitations led to declining BNCT interest in North America and Europe. However, Japanese investigators revitalized the field in the 1980s and 1990s with the introduction of boronophenylalanine (BPA) and sodium borocaptate (BSH), which demonstrated significantly improved tumor selectivity, promoting renewed interest in gliomas and head and neck cancers [19,20]. Recently, a major paradigm was promoted by the development of hospital-compatible accelerator-based epithermal neutron sources, eliminating reliance on nuclear reactors and enabling broader clinical adoption [21]. This technological transition, combined with advances in boron carrier chemistry, image-guided treatment planning, and biologically weighted BNCT dosimetry, reignited interest. Presently, BNCT is approved in Japan for treating unresectable, recurrent head and neck cancer [22]. A summary of currently active accelerator-based BNCT centers, their neutron-source technologies, and clinical implementation status is provided in Table 1.

## 4. Workflow

Patient selection is based on histopathologic confirmation, prior treatments, performance status, and anatomical suitability for neutron irradiation. In the re-irradiation setting, a minimum interval of approximately three months from prior radiotherapy is recommended, along with adequate hematologic parameters and the absence of uncontrolled edema or mass effect. Tumor location, depth, association with skull-base anatomy, and proximity to critical structures are also key determinants of eligibility and achievable dose. A boron-containing compound is administered intravenously. Quantitative ^18^F-BPA PET is subsequently performed to assess the tumor-to-normal tissue and tumor-to-blood boron concentration ratios, which guide treatment candidacy and inform estimation of the optimal irradiation time window. These pharmacokinetic data are incorporated into image-based treatment planning, with MRI and CT datasets co-registered to PET for accurate delineation of target volumes and organs at risk.

Monte Carlo-based treatment planning systems are used to simulate neutron transport and model the individual contributions of the ^10^B(n,α)^7^Li reaction, proton recoil, nitrogen capture, and gamma components. Contemporary platforms increasingly support iterative dose optimization, enabling refinement of beam geometry, irradiation time, and dose constraints to maximize compound biological effectiveness (CBE)- and relative biological effectiveness (RBE)-weighted tumor dose.

Accurate and reproducible patient positioning is a critical determinant of treatment accuracy in BNCT, given the steep dose gradients and dependence on neutron beam geometry. Modern accelerator-based BNCT facilities employ six-axis robotic positioning systems integrated with treatment planning and image-guidance workflows, achieving millimeter-level accuracy, with reported maximum deviations of approximately 2–3 mm in source-to-skin distance and along the treatment surface [23,24,25]. Recent advances in robotic platforms further expanded positioning capabilities, allowing flexible patient orientations, including non-supine and seated treatment positions, to accommodate challenging tumor locations and patient-specific anatomical constraints. Seated positioning has been explored to improve patient comfort, facilitate stable head fixation, and enable more favorable neutron beam geometry, especially in head and neck applications [26,27]. In parallel, surface-guided positioning approaches combining binocular stereo-vision systems with closed-loop robotic corrections demonstrate reliable millimeter-scale accuracy in phantom validation studies. These developments highlight the growing versatility of robotic positioning systems and their potential to provide increasingly individualized treatment plans.

Advances in real-time in vivo dosimetry are an active area of BNCT research. Experimental systems, including i-TED Compton camera arrays, are being developed to detect and image the 478 keV prompt gamma rays emitted during the ^10^B(n,α)^7^Li reaction [28,29,30]. Additional approaches, such as prompt gamma-SPECT and silicon/CdTe-based Compton cameras, demonstrated feasibility in preclinical and experimental settings for noninvasive monitoring of boron neutron capture reactions.

## 5. Boron Delivery Agents

### 5.1. First Generation

Early BNCT research in the 1950s–1960s at Brookhaven National Laboratory and the MGH/MIT reactor programs evaluated simple inorganic boron compounds including sodium borate formulations and sodium decahydrodecaborate (Na_2_B_10_H_10_) as potential boron carriers. These first-generation compounds exhibited rapid systemic clearance, poor tumor-to-normal tissue concentration ratios, and minimal selective tumor uptake, resulting in unfavorable biodistribution prohibitive of providing a therapeutic benefit, contributing to the discontinuation of U.S. clinical trials [14,31]. In contrast, subsequent Japanese programs advanced more chemically sophisticated boron cluster compounds, including BSH, which demonstrated improved biological behavior suitable for clinical BNCT [32].

### 5.2. Second Generation

BSH and BPA possessed improved tumor selectivity and higher boron delivery. BSH was the first boron carrier to produce measurable therapeutic benefit in GBM patients [33]. Its high boron density, passive penetration into regions of blood–brain barrier disruption, and distribution throughout the infiltrative extracellular tumor compartment enabled significantly higher intratumoral boron levels (19.9 +/− 9.1 ppm) [34]. However, BSH’s major limitation was insufficient intracellular penetration and only modest tumor-to-normal selectivity. These limitations were addressed by BPA, which exploits the L-type amino acid transporter 1 (LAT1) to achieve active, energy-dependent intracellular uptake, thereby delivering substantially higher boron concentrations directly into tumor cells, producing a more favorable tumor-to-normal tissue ratio [35]. Clinically, BPA is administered as a water-soluble complex with fructose (BPA-f), which significantly improves intravenous delivery and enables the high infusion doses required for BNCT. In addition to its therapeutic role, the radiolabeled analog ^18^F-BPA serves as a theranostic imaging agent, allowing non-invasive PET assessment of tumor boron uptake, patient selection, and treatment planning. Emerging analogs such as boronotyrosine aim to further improve tumor uptake, retention, and tumor-to-normal tissue ratios. The complementary biological profiles of BPA and BSH, BPA providing high intracellular tumor targeting, and BSH distributing within the extracellular and infiltrative compartments, form the rationale for combined administration [36,37,38]. This strategy improves the spatial homogeneity of boron delivery across heterogeneous glioma tissue and has the potential to optimize BNCT dose effectiveness.

### 5.3. Third Generation

Third-generation boron delivery agents represent a significant advance in BNCT, leveraging contemporary molecular engineering and nanotechnology to overcome the limited tumor selectivity and suboptimal boron accumulation of earlier compounds like BPA and BSH. These multifunctional platforms integrate tumor-targeting ligands, diagnostic imaging moieties, and high-capacity boron clusters within a single construct, enabling markedly enhanced tumor specificity and more uniform intratumoral boron distribution. Ongoing preclinical development includes antibody-boron conjugates, boron-enriched liposomes and nanoparticles, peptide-targeted boron conjugates, and novel small-molecule carriers, each demonstrating promising potential to increase boron delivery to CNS malignancies [39].

Antibody-boron conjugates (ABCs) represent an emerging third-generation BNCT strategy, combining antigen-specific molecular targeting with substantially higher boron payload capacity than small-molecule carriers [40,41]. HER2-directed boron-loaded immunoliposomes have demonstrated selective tumor uptake and markedly enhanced BNCT cytotoxicity in both in vitro and xenograft models, achieving up to 14-fold greater therapeutic activity compared with the clinical BPA-fructose complex in HER2-overexpressing ovarian cancer cells [42,43]. Similarly, EGFR- and LDLR-targeted antibody-functionalized boron carbide nanoparticles have shown approximately two-fold increased cellular association in receptor-expressing tumor cells, with intracellular boron concentrations reaching 9.58 ± 2.6 mg/L per million cells in high-expressing lines. In parallel, Fc-binding peptide–based targeting systems, such as Z33 peptide–dodecaborate conjugates, have demonstrated effective antibody-mediated, receptor-dependent boron delivery [44]. To date, all ABC research remains preclinical, with no clinical trials currently reported.

Peptide-conjugated boron delivery systems constitute a versatile third-generation BNCT platform designed to enhance tumor specificity and intracellular boron accumulation [45,46]. By coupling boron-rich clusters to peptides that recognize receptors or surface markers overexpressed on cancer cells, these constructs enable active molecular targeting and receptor-mediated endocytosis [46]. Several peptide classes have shown promise: Fc-binding peptides (Z33) for antibody-based targeting, tumor-vasculature-targeted peptides (SP94) achieving tumor-to-blood ratios of 5.92 ± 0.45, receptor-directed ligands such as GRPR agonists with up to 80 boron atoms per molecule while maintaining receptor activation, neuropeptide Y analogs targeting hY1R with maximized carborane loading, ghrelin receptor (GHSR)-targeted peptide conjugates demonstrating receptor-mediated tumor uptake, and cell-penetrating peptides such as octa-arginine (R8), TAT, or angiopep-2 [44,47,48,49]. The angiopep-2 conjugates achieved 86.5% clonogenic cell death in vitro and 62.9% tumor shrinkage in intracranial glioma models, significantly outperforming BPA [49]. GHSR-targeted boron delivery systems represent another emerging peptide-based strategy. GHSR is overexpressed in several malignancies, including glioma, and provides a molecular entry route for receptor-mediated internalization [50,51,52]. Carborane-functionalized ghrelin analogues and related peptide conjugates have demonstrated selective tumor cell uptake and enhanced BNCT cytotoxicity in preclinical models, supporting the feasibility of exploiting the ghrelin axis for targeted boron delivery. These systems demonstrate enhanced cellular uptake, higher intratumoral boron concentrations, and superior BNCT therapeutic efficacy across multiple preclinical models.

Boronated nucleosides and DNA-intercalating compounds were developed to achieve nuclear localization and mitotic trapping, thereby maximizing DNA damage from the ^10^B(n,α)^7^Li reaction at its most critical target [53]. Carboranyl thymidine analogs, particularly N5-2OH, demonstrated selective retention by thymidine kinase-1-positive tumors with tumor-to-brain ratios of 8.5 following convection-enhanced delivery. Nucleotide borate esters, such as ATP borate ester, exhibited significantly high radiosensitization values, with tumor boron concentrations comparable to BPA’s [54]. Although in vitro studies demonstrated substantial cytotoxic potential, unfavorable in vivo pharmacokinetics, rapid systemic clearance, and dose-limiting toxicities largely impeded further development [55].

Nanocarrier-based platforms provide an opportunity to deliver high boron payloads with tunable biodistribution, including boron-functionalized nanodiamond constructs and polymer-based nanoparticle systems designed for active tumor targeting [56,57,58]. PEGylated liposomes encapsulating nido-carborane or BSH have shown prolonged circulation times and improved tumor retention, and their further functionalization with folate, transferrin, or other tumor-targeting ligands is under active investigation [56]. Carboranyl-phosphatidylcholine-based liposomes (boronosomes) demonstrated high tumor accumulation and long retention with clear irradiation backgrounds on PET imaging, with further improvements when combined with chemotherapy agents, particularly PARP1 inhibitors. Gadolinium-boron integrated lipid nanocarriers with Angiopep-2 modification enabled both targeted delivery and MRI-guided BNCT, significantly prolonging survival in orthotopic GBM models [59]. Additional nanoformulations including polymeric micelles, dendrimers such as PAMAM, mesoporous silica nanoparticles, and metallacarborane hybrids have demonstrated the capacity to deliver very high boron loads while enabling multivalent tumor targeting and enhanced intracellular uptake [59,60,61]. Although these nanotechnologies show strong preclinical promise, clinical translation will require rigorous standardization, including robust boron-specific dosimetry, comprehensive toxicologic evaluation, and scalable manufacturing quality control.

Recent developments include novel transporter-targeted small molecules that expand BNCT beyond LAT1-dependent BPA. GluB-2, an ASCT2-targeted carrier, achieves tumor boron concentrations exceeding 20 µg/g after both intravenous and intraperitoneal administration, representing the first non-BPA small-molecule carrier to reach therapeutic thresholds, with pronounced tumor suppression in BPA-refractory U87MG xenograft models [62]. Boronotyrosine, a borylated tyrosine analog, demonstrated 2–3 times greater boron delivery in vivo than BPA with superior tumor-to-blood ratios and up to 4 times greater cellular uptake in vitro [63]. Folate receptor-targeted PBC-IP administered via convection-enhanced delivery showed significantly improved survival compared to BPA alone in F98 rat glioma models, with long-term survivors observed only in PBC-IP-treated groups [64].

A concise overview of first-, second-, and emerging third-generation boron delivery strategies including their uptake mechanisms, advantages, and limitations is provided in Table 2.

## 6. Neutron Sources for BNCT

Historically, reactor-based BNCT relied on nuclear research reactors to generate neutron beams through fission processes. Although these systems provided high neutron flux, they required specialized infrastructure, extensive shielding, and access to dedicated reactor facilities, which limited clinical scalability and regulatory feasibility [12]. In contrast, modern accelerator-based BNCT systems generate neutrons using compact proton accelerators directed at lithium or beryllium targets via the ^7^Li(p,n)^7^Be or Be(p,n) reactions [12,25]. The resulting neutrons are subsequently moderated using beam-shaping assemblies to achieve the epithermal energy range optimal for tissue penetration and tumor targeting [28]. These hospital-compatible platforms enable installation within medical centers and support standardized treatment planning, dosimetry, and regulatory integration [65]. Although both approaches rely on the same ^10^B(n,α)^7^Li reaction for tumor cell kill, accelerator-based systems represent a major translational advance that has enabled broader clinical implementation. Notably, Japan’s regulatory approval in 2020 of accelerator-based BNCT for unresectable locally advanced or locally recurrent head and neck cancer marked the first worldwide approval of an accelerator-based BNCT system as a medical device.

Broader clinical adoption of BNCT will depend on the continued deployment and standardization of hospital-based accelerator neutron sources. Compact accelerator systems are increasingly favored due to their reduced physical footprint, lower infrastructure requirements, and feasibility for installation within hospitals [66,67,68]. These platforms typically generate epithermal neutrons using low-energy proton or deuteron beams incident on lithium or beryllium targets, with beam-shaping assemblies optimized to meet International Atomic Energy Agency (IAEA) recommendations [25,69]. Modern designs incorporate modular collimator systems allowing diameter adjustment from 8 to 17 cm to accommodate different tumor sizes and positions, with larger beam diameters delivering higher dose rates and deeper penetration. Recent innovations include dual-port designs with both thermal and epithermal neutron ports for treating superficial and deep-seated tumors, and novel BSA-free approaches using near-threshold proton reactions that may simplify facility design and enable energy-controlled neutron penetration depth [70]. Neutron flux optimization is achieved through careful selection of target materials and beam current parameters, with current systems generating epithermal neutron fluxes of 10^8^–10^9^ n/cm^2^/s at treatment positions. Standardization of neutron spectra and dose-calculation methodologies across these platforms will be critical for multi-institutional trials.

## 7. BNCT Compared with Proton and Carbon Ion Radiotherapy

A key distinction between BNCT and other particle therapies lies in the quality, magnitude, and spatial distribution of LET. Proton beams are predominantly low-LET radiation, with mean LET values of approximately 0.5–2 keV/µm, increasing modestly near the Bragg peak. Their relative biological effectiveness (RBE) is typically ~1.1, only slightly greater than photons; however, experimental and modeling studies suggest that RBE may increase toward the distal edge of the Bragg peak, with potential implications for treatment planning and normal tissue toxicity. Thus, the clinical advantages of proton therapy arise primarily from superior physical dose conformality, rather than enhanced radiobiological potency [71]. Carbon ions are high-LET particles, with LET values typically ranging from 40 to 80 keV/µm at the Bragg peak and exceeding 100 keV/µm under certain beam and tissue conditions. Their RBE generally ranges from 2 to 3, depending on tissue type, dose per fraction, and biological endpoint, and reflects dense ionization tracks that induce complex, difficult-to-repair DNA double-strand breaks and enhanced cell-killing efficiency; however, in clinical practice, these theoretical radiobiological advantages have not consistently translated into clearly superior outcomes compared with other advanced radiation modalities [72].

BNCT delivers a pharmacologically localized form of ultra-high LET irradiation. Its microdosimetric precision represents both the principal strength and central challenge of BNCT, as therapeutic efficacy depends critically on selective boron delivery, intracellular uptake, and uniform intratumoral distribution. In contrast, proton and carbon-ion therapies rely primarily on physical dose distribution, with biological effectiveness governed by intrinsic beam properties rather than pharmacologic targeting. A comparative overview of proton therapy, carbon-ion therapy, and BNCT including LET characteristics, RBE, spatial dose behavior, and mechanisms of selectivity is summarized in Table 3.

## 8. BNCT for CNS Tumors

BNCT applications in the CNS were driven by the need to for options to treat intrinsically radioresistant tumors or upon exhausting conventional therapeutic options. Consequently, early reactor-based clinical experiences were largely confined to high-grade gliomas (HGGs) in the salvage setting, where standard re-irradiation strategies offered limited efficacy and carried substantial risks of normal tissue toxicity. As BNCT technology evolved particularly with the transition to accelerator-based neutron sources, its clinical application expanded beyond salvage therapy.

### 8.1. Recurrent GBM

Reactor-based BNCT for recurrent GBM was first systematically evaluated in a Swedish cohort of 12 patients treated with a 6 h high-dose BPA infusion, demonstrating median OS of 22.2 months from initial diagnosis, 13.7 months from recurrence, and 8.7 months from BNCT, with preservation of quality of life until shortly before progression and no grade 3–4 acute toxicities [73]. The Osaka group subsequently reported 22 patients with recurrent malignant glioma (19 GBM) treated with BPA and/or BSH, achieving median OS after BNCT of 10.8 months for the full cohort and 9.6 months for recurrent GBM. Among high-risk RPA classes, median OS reached 9.1 months, compared with 4.4 months in the Carson et al. series, suggesting a survival advantage despite the absence of a contemporaneous control arm [74,75].

Accelerator-based BNCT was evaluated in the multicenter phase II JG002 trial, which treated 27 recurrent malignant gliomas (24 GBM) with cyclotron-based SPM-011 (500 mg/kg BPA over 3 h) [76,77]. Extended follow-up data published in 2025 demonstrated a 1-year OS of 79.2% (95% CI: 63.3–88.7) and median OS of 19.2 months (95% CI: 13.1–24.8) in the GBM subset, with 2-year and 3-year OS rates of 33.3% and 20.8%, respectively [78]. This represents substantially higher survival than the 34.5% 1-year OS and 10.5-month median OS reported in the Japanese bevacizumab trial JO22506 [76]. To address concerns about radiation necrosis and pseudoprogression following high-LET re-irradiation, Furuse et al. combined reactor-based BNCT with early, successive bevacizumab (10 mg/kg initiated 1–4 weeks post-BNCT) in 25 patients with recurrent malignant glioma. In the GBM subgroup (n = 14), median OS was 21.4 months (95% CI: 7.0–36.7) and PFS 8.3 months (95% CI: 4.2–12.1), with no cases of pseudoprogression or radiographic radiation necrosis observed during bevacizumab treatment. Grade ≥3 adverse events occurred in six patients [79].

Overall, reactor- and accelerator-based experiences suggests BNCT is technically feasible, generally tolerable, and capable of producing encouraging survival outcomes in recurrent GBM, with median OS after treatment ranging from 8 to 10 months in earlier cohorts to ~19–21 months in more recent, highly selected populations treated with BNCT and/or bevacizumab. However, these findings are derived from non-randomized studies with heterogeneous prior therapies, variable performance status, and frequent bevacizumab use.

### 8.2. Newly Diagnosed GBM

In a prospective study by Yamamoto et al., 15 patients undergoing debulking surgery followed by single-fraction BPA/BSH-mediated BNCT achieved a median OS of 25.7 months and 1- and 2-year OS rates of 80.0% and 53.3%, respectively. Median time to progression was approximately 12 months, and treatment was generally well tolerated; however, one patient who received frontotemporal irradiation without optimal neutron shielding developed Grade 4 post-epileptic brain swelling requiring surgical intervention [80,81]. Kageji et al. treated 23 newly diagnosed GBM patients using either intraoperative BSH-BNCT or external-beam BNCT with BPA + BSH, reporting a median 19.5 month OS and 2-, 3- and 5-year OS rates of 31.8%, 22.7% and 9.1% with no treatment-limiting adverse events [82]. The Osaka group reported 21 newly diagnosed GBM patients treated with BNCT entailing simultaneous BPA and BSH administration, with median OS of 15.6 months overall and 23.5 months for those receiving BNCT followed by 20–30 Gy external beam radiotherapy [80].

The Studsvik Phase II trial evaluated a prolonged 6 h infusion of BPA-f in 29 newly diagnosed GBM patients. Median OS was 17.6 months from surgery, with a 1-year OS rate of 78.7%, with only 13–14% of patients experiencing severe adverse events. Retrospective comparison using RPA stratification suggested BNCT may achieve outcomes comparable to standard chemoradiation, with greater relative benefit in MGMT-unmethylated tumors (adjusted HR vs. RT/TMZ ≈ 0.68) [83,84].

Accelerator-based BNCT for newly diagnosed GBM is being evaluated in combination with standard therapy. The University of Tsukuba TB-GB-01 trial is the first Phase I study evaluating the combination of accelerator-based BNCT, external beam radiation (40 Gy in 20 fractions), and temozolomide in newly diagnosed GBM [85]. This trial uses dose escalation starting with a BNCT normal brain maximum dose of 7 Gy at D2cc, with the primary endpoint being dose-limiting toxicities and secondary endpoints including adverse event rates, treatment completion rate, response rate, PFS, and OS.

While limited by small cohorts and heterogeneous treatment protocols, these non-randomized institutional experiences support BNCT monotherapy achieving survival outcomes comparable to standard chemoradiation.

### 8.3. Pediatric HGGs

Clinical experience with BNCT in pediatric high-grade CNS tumors remains limited; Nakagawa et al. treated 23 children younger than 15 years (including 4 patients under 3 years) with malignant gliomas, including GBM, anaplastic astrocytoma, anaplastic ependymoma, and primitive neuroectodermal tumor, using BPA- or BSH-mediated BNCT. BNCT achieved local tumor control across nearly all histologies; however, children with GBM and PNET ultimately died from cerebrospinal fluid and/or CNS dissemination rather than local failure. In contrast, four of six children with anaplastic astrocytoma and one with anaplastic ependymoma remained alive without recurrence. Notably, BNCT was delivered without treatment-limiting acute or late toxicities, long-term survivors maintained preserved neurological function and normal educational development [86].

Chen et al. reported a series in five children with recurrent, previously irradiated GBM, who collectively underwent nine BNCT treatments to ten tumor sites. With a median follow-up of 5.3 months, objective responses were observed in majority lesions (one complete response and three partial responses). The median PFS was 3.8 months, and no severe radiation necrosis or serious BNCT-related complications were noted [87]. Huang et al. treated six children with recurrent, previously irradiated diffuse midline gliomas who received two fractions of BPA-based BNCT. BNCT produced partial radiographic responses in three patients and stable disease in the remaining three with mean post-BNCT OS of 6.39 months and 4.35 months PFS. Treatment was generally well tolerated, with only one case of radiation necrosis in a child who received prior hypofractionated radiotherapy [88].

These pediatric experiences collectively demonstrate BNCT promotes local tumor control with minimal toxicity in carefully selected pediatric patients and could be considered for localized treatment and/or gross tumor/tumor bed boost in conjunction with modalities focused on CSF-dissemination. This approach is particularly rational in a population where conventional high-dose radiotherapy is often avoided because of the risk of severe neurocognitive sequelae.

### 8.4. Meningiomas

Early translational studies demonstrated meningiomas accumulate therapeutically meaningful intratumoral boron concentrations with both BSH and BPA [89,90]. The first reported clinical case entailed a patient wheelchair-bound due to a recurrent WHO grade 3 papillary meningioma regaining ambulation within a week of BPA + BSH BNCT, with the treated lesion regressing by 50% at 26 weeks. A subsequent BNCT session targeting a second contralateral lesion with high ^18^F-BPA uptake produced similar radiographic regression [91]. Subsequent reactor-based series from Osaka Medical College expanded these observations to larger cohorts of recurrent WHO grade 2–3 or malignant meningiomas refractory to surgery and conventional radiotherapy [92,93,94]. In series of Miyatake two of three anaplastic meningiomas achieved complete response, all six patients with follow-up imaging showed radiographic improvement, and pre-treatment neurological symptoms such as hemiparesis and facial pain improved in all but one patient [94]. Kawabata et al. reported a mean tumor-volume reduction of 64.5% at 2 months in 20 patients with recurrent high-grade meningioma treated with BPA-based BNCT, further supporting the capacity of BNCT to induce substantial, and often rapid response in this otherwise treatment-refractory population [92]. Takai and colleagues from Osaka retrospectively reviewed 44 patients with recurrent WHO grade 2–3 meningioma treated with BPA-based BNCT with or without BSH between 2005 and 2019 [95]. The median OS after BNCT was 29.6 months (95% CI: 16.1–40.4), with grade-specific outcomes showing median OS of 44.4 months for grade 2 versus 21.55 months for grade 3 disease (*p* = 0.0009). Follow-up images obtained from 36 cases showed tumor shrinkage in 35 patients during the observation period, occasionally following a short period of pseudoprogression. The median PFS after BNCT was 13.7 months (95% CI: 8.3–28.6), with significantly better outcomes for grade 2 (24.3 months) versus grade 3 (9.4 months) meningiomas (*p* = 0.0024).

A Taiwanese single-center series of 13 previously irradiated recurrent grade 1–3 meningioma patients (treated with salvage BNCT between 2020 and 2024 associated dose as a critical determinant of treatment response [90]. In this cohort, 5 of 13 patients (38%) met RANO criteria for partial or complete response, with responders receiving a significantly higher mean tumor dose (45.10 GyE vs. 25.85 GyE) than non-responders. Non-skull base tumors, amenable to safely receiving higher BNCT doses due to more favorable beam geometry and greater distance from critical structures, trending toward improved response [96]. No severe acute or late toxicities were reported. This study also introduced the novel use of ^18^F-Fluciclovine PET as an alternative to ^18^F-BPA PET for tumor uptake assessment, expanding imaging options for BNCT planning. A recent case report from Taiwan demonstrated successful salvage BNCT for a WHO grade 3 malignant meningioma with skull bone and scalp invasion, achieving near-total regression with two courses of BNCT (mean tumor doses 41.52 and 50.22 GyE) without significant toxicity [97].

In the phase II jRCT2051190044 study published in January 2026, Kashiwagi and colleagues randomized patients with refractory recurrent WHO grade 2–3 meningiomas after prior photon radiotherapy to BNCT plus best supportive care (BSC) versus BSC alone (2:1 allocation; 18 patients analyzed) [98]. The trial met its primary endpoint: independently assessed median PFS was 14.4 months in the BNCT arm versus 1.4 months in the BSC arm (95% CI: 7.9–26.4 vs. 1.0–9.0; *p* = 0.0157), and investigator-assessed PFS showed similar benefits (14.7 vs. 1.5 months, *p* = 0.0002). The objective response rate in the BNCT arm was 27.3%. One- and two-year OS rates in the BNCT arm approached 100% and 90%, respectively, although OS interpretation is limited by cross-over to “rescue BNCT” in five of six control patients. Safety findings were consistent with earlier series, with predominantly manageable alopecia and radiation-related edema.

Taken together, that the aforementioned studies support BPA/BSH-based BNCT biological activity and clinically utility in heavily pretreated grade 2–3 meningiomas. To provide a structured overview of the current clinical evidence base, published and ongoing clinical studies of BNCT in central nervous system tumors are summarized in Table 4.

## 9. Limitations

The majority of clinical BNCT data entail single-arm, non-randomized studies with small cohorts. These heterogeneous series often include patients with diverse tumor histologies, variable prior treatments, inconsistent boron-carrier protocols (BPA, BSH, or combinations), and evolving neutron-source technologies (reactor- vs. accelerator-based systems), limiting the ability to draw comparative conclusions. Interpretation of survival outcomes is confounded by frequent use of concurrent or sequential therapies, particularly bevacizumab, which can substantially alter PFS, reduce radiation-related edema, and obscure attribution of treatment effects. Additionally, boron pharmacokinetics, intratumoral distribution, and cellular microheterogeneity remain major sources of biological variability. Most clinical reports lack standardized quantification of boron concentration or spatial distribution, and differences in infusion protocols, PET imaging, and boron carriers complicate cross-study comparison. These factors are especially relevant for infiltrative gliomas, where non-uniform boron uptake may limit therapeutic coverage. Methodological inconsistencies, including variable definitions of progression, differences in imaging follow-up protocols, and the lack of centralized radiologic review, impair the reliability of PFS and response assessments. High-LET-related phenomena such as pseudoprogression and radiation-induced edema further complicate radiographic interpretation. Finally, long-term toxicity data, particularly for AB-BNCT and pediatric patients, remain sparse. Although early reports suggest favorable neurocognitive profiles and acceptable safety, follow-up durations are short, and late effects including radionecrosis, neurocognitive decline, or vascular injury cannot yet be fully characterized.

## 10. Conclusions

BNCT has progressed substantially from its early reactor-based origins where limited boron selectivity and suboptimal neutron spectra constrained therapeutic benefit to an increasingly refined, clinically feasible modality enabled by modern accelerator technology. Current clinical data, particularly from phase II trials and large institutional series, demonstrate encouraging survival outcomes in recurrent HGGs and high-grade meningiomas, with a favorable safety profile despite prior irradiation in most patients. In this context, BNCT appears particularly well suited as a salvage strategy for patients with limited remaining local treatment options, where its pharmacologically localized, ultra-high LET mechanism may offer meaningful cytoreduction. Beyond salvage, emerging exploratory analyses suggest that BNCT may hold a niche role in biologically aggressive subgroups, including MGMT-unmethylated glioblastoma, in which resistance to alkylating chemotherapy limits the efficacy of standard chemoradiation. While BPA remains the cornerstone boron carrier in current clinical practice, ongoing advances in boron-carrier chemistry, including third-generation antibody, peptide, and nanocarrier platforms, offer the potential to improve tumor specificity and overcome intratumoral heterogeneity. In parallel, the expanding availability of hospital-compatible accelerator-based BNCT systems is expected to improve accessibility, standardize treatment workflows, and facilitate multicenter collaboration. Ultimately, definitive integration of BNCT into the neuro-oncology treatment paradigm will require prospective, randomized clinical trials to clarify its comparative efficacy, optimal sequencing, and patient selection relative to established radiotherapy and systemic approaches.

## Figures and Tables

**Table 1:** Operational accelerator-based BNCT centers worldwide, including neutron source type and current clinical status.

Center	Country	Source Type (Accelerator + Target)	Neutron Energy	Current Status
Kansai BNCT Medical Center (Osaka Medical and Pharmaceutical University)	Japan	Cyclotron (HM-30, Sumitomo Heavy Industries) → Be	~30 MeV	Routine clinical treatment; BNCT approved in Japan (2020) and reimbursed by National Health Insurance
Southern Tohoku BNCT Research Center (NeuCure BNCT30)	Japan	Cyclotron (HM-30) → Be	~30 MeV	Routine clinical treatment; active treatment of head and neck cancer and other malignancies
National Cancer Center Hospital -Division of BNCT Medical Research	Japan	RFQ Accelerator → solid Li	~2.5 MeV	Phase I/II clinical trials; vertical downward neutron beam configuration
University of Tsukuba Hospital	Japan	Accelerator-based system → Li	~2.5 MeV	Phase I clinical trials, including newly diagnosed GBM
Helsinki University Hospital	Finland	Electrostatic Cockcroft–Walton proton accelerator (nuBeam) → Li	~2.6 MeV (≈30 mA)	Clinical trials; first European accelerator-based BNCT facility
Xiamen Humanity Hospital	China	Tandem electrostatic accelerator (NeuPex platform) → Li	~2.5 MeV	Early clinical research/limited patient treatments reported; peer-reviewed data remain limited

**Table 2:** Representative boron delivery compounds and nanoplatforms evaluated for boron neutron capture therapy (BNCT), summarizing their mechanisms of tumor uptake, key advantages, and current limitations or developmental status.

Compound/Platform	Mechanism of Tumor Uptake	Advantages	Limitations/Status
First generation			
Borax, simple boron salts	Passive, non-specific distribution	Proof of concept in early trials	Poor tumor selectivity; systemic toxicity; abandoned
Sodium decahydrodecaborate (Na_2_B_10_H_10_)	Non-specific distribution	High boron content	Unfavorable biodistribution; discontinued
Second generation			
BSH (sodium borocaptate)	Predominantly extracellular; accumulation in BBB-disrupted regions	High boron density; clinical experience	Limited intracellular uptake; modest tumor:normal ratio
BPA (boronophenylalanine; BPA-fructose)	LAT1 transporter-mediated cellular uptake; PET-quantifiable	Gold standard; intracellular delivery; supports ^18^F-BPA PET planning	Heterogeneous distribution; LAT1 dependence
Third generation (preclinical/translational)			
Boronated nucleosides/DNA-intercalators	Nuclear targeting; mitotic trapping	Direct DNA targeting; high biological effectiveness	Preclinical only; toxicity/dosimetry under investigation
Antibody-boron conjugates (ABC)	Antigen-specific binding (e.g., EGFR, HER2)	High specificity; large boron payload	Complex synthesis; immunogenicity; preclinical only
Antibody-functionalized nanoparticles/immunoliposomes	Receptor-mediated uptake via antibody targeting	Combines high payload with active targeting	Tumor penetration heterogeneity; preclinical only
Peptide-conjugated boron clusters (e.g., GRPR, hY1R)	Peptide ligands for tumor cell surface receptors	Enhanced selectivity; modular design	Preclinical only; pharmacokinetics not yet standardized, variable receptor expression
Tumor vasculature-targeted peptides (e.g., SP94, Annexin A1)	Vascular/microenvironment targeting	High tumor–blood ratios in models	Target heterogeneity; preclinical only
Cell-penetrating/BBB-penetrating peptides (R8, TAT, Angiopep-2)	Cellular penetration or transcytosis	Improved intracellular and CNS delivery	Safety optimization needed
Boron-rich liposomes/nanoparticles	Enhanced permeability and retention (EPR); targeted modifications (e.g., folate, transferrin)	High boron payload; potential for controlled release	Heterogeneous tumor penetration; clinical translation pending
Boronosomes (carboranyl lipid vesicles)	Lipid-based tumor accumulation	Long tumor retention; imaging compatibility	Heterogeneous penetration; preclinical only
Theranostic Gd-B nanocarriers	Targeted delivery with MRI guidance	Image-guided BNCT potential	Complex design; regulatory hurdles
Transporter-targeted small molecules (e.g., GluB-2/ASCT2)	Alternative amino-acid transporter uptake	Active in BPA-refractory models	Early-stage; preclinical only

**Table 3:** Comparison of proton therapy, carbon-ion therapy, and boron neutron capture therapy (BNCT) with respect to LET, RBE, range, mechanisms of selectivity, and key radiobiological features.

Modality	Proton Therapy	Carbon-Ion Therapy	Boron Neutron Capture Therapy
LET (keV/µm)	~0.5–2 (low-LET; modest rise at Bragg peak)	~50–80 at Bragg peak (high-LET)	~150 (ultra–high LET for α and ^7^Li)
RBE	~1.1	2–3 (tissue/endpoint dependent)	Variable; high (effective only within boron-containing cells)
Range in tissue	Several cm (beam penetration; depth dose conformality)	Several cm (Bragg peak range)	5–9 µm (cellular diameter)
Mechanism of selectivity	Physical dose distribution (Bragg peak)	Physical + radiobiological (high-LET effect in target volume)	Biological selectivity via boron compound uptake
Key Features	Conformal dose shaping; no significant intrinsic radiobiological advantage	Dense ionization, complex DNA damage; effective in radioresistant tumors	Ultra-localized cell kill; efficacy determined by boron delivery and microdistribution

**Table 4:** Selected clinical studies of BNCT in CNS tumors (published and ongoing).

Study	Design	Population	N	Boron Agent	Neutron Source	Key Outcomes
Yamamoto et al. [80,81]	Pilot study (2 protocols)	Newly diagnosed GBM	15	BSH (all); BSH + BPA (protocol-2 only)	Reactor (epithermal)	Median OS 25.7 mo; TTP 11.9 mo; 1-year OS 80%, 2-year OS 53.3%
Kageji et al. [82]	Retrospective	GBM (newly diagnosed and recurrent)	23	BSH ± BPA	Reactor	Median OS 19.5 mo; 2-year OS 26.1%; 5-year OS 5.8%
Sköld et al. (Studsvik) [83,84]	Phase II	Newly diagnosed GBM	29	BPA-fructose (6-hr infusion)	Reactor (epithermal)	Median OS 17.6 mo; comparable to RT alone; possible advantage in MGMT unmethylated
TB-GB-01 (Tsukuba) [85]	Phase I (ongoing)	Newly diagnosed GBM	12–18 (target)	BPA + EBRT (40 Gy) + TMZ	Accelerator	Dose-escalation; primary endpoint DLT
Miyatake et al. [74]	Retrospective	Recurrent malignant glioma	22 (19 GBM)	BPA ± BSH	Reactor (epithermal)	Median OS 10.8 mo (all MG); 9.6 mo (GBM); High-risk RPA 9.1 mo vs. 4.4 mo historical
JG002 [76,78]	Phase II	Recurrent GBM	24	BPA	Accelerator (cyclotron)	Median OS 19.2 mo; 1-year OS 79.2%; 2-year OS 33.3%; 3-year OS 20.8%
Furuse et al. [79]	Retrospective	Recurrent malignant glioma	25 (14 primary GBM, 11 non-primary)	BPA + BSH	Reactor	Primary GBM: median OS 21.4 mo, PFS 8.3 mo; non-primary: median OS 73.6 mo, PFS 15.6 mo
Huang et al. [88]	Case series	Recurrent pediatric DMG	6	BPA	Reactor (Tsing Hua)	3 PR, 3 SD; OS 6.39 mo; PFS 4.35 mo; low toxicity
Osaka Medical College series [95]	Retrospective	Recurrent/refractory HGM	46	BPA ± BSH	Reactor	Favorable outcomes in refractory cases
Lan et al. [90]	Retrospective feasibility	Recurrent meningioma (1 grade 3, 6 grade 2, 6 grade 1)	13	BPA	Reactor (Tsing Hua)	Response rate 38%; responders had higher tumor dose (45.10 vs. 25.85 GyE, *p* = 0.003)
Kashiwagi et al. [98]	Phase II RCT (2:1)	Recurrent HGM after RT	18 (12 BNCT, 6 control)	BPA	Accelerator (cyclotron)	Median PFS 14.4 mo (BNCT) vs. 1.4 mo (control), *p* = 0.0157; ORR 27.3%

BNCT, boron neutron capture therapy; GBM, glioblastoma; HGM, high-grade meningioma; DMG, diffuse midline glioma; BPA, boronophenylalanine; BSH, sodium borocaptate; EBRT, external beam radiotherapy; TMZ, temozolomide; OS, overall survival; PFS, progression-free survival; TTP, time to progression; PR, partial response; SD, stable disease; RPA, recursive partitioning analysis; DLT, dose-limiting toxicity; RT, radiotherapy; MGMT, O^6^-methylguanine-DNA methyltransferase; mo, months.

## Data Availability

No new data were created or analyzed in this study. Data sharing is not applicable to this article.

## Abbreviations

BNCT—Boron Neutron Capture Therapy, GBM—Glioblastoma, HGG—High-Grade Glioma, HGM—High-Grade Meningioma, CNS—Central Nervous System, LET—Linear Energy Transfer, RBE—Relative Biological Effectiveness, CBE—Compound Biological Effectiveness, BPA—Boronophenylalanine, BSH—Sodium Borocaptate, LAT1—L-type Amino Acid Transporter 1, GHSR—Growth Hormone Secretagogue Receptor, GRPR—Gastrin-Releasing Peptide Receptor, hY1R—Human Neuropeptide Y Receptor 1, EBRT—External Beam Radiotherapy, TMZ—Temozolomide, OS—Overall Survival, PFS—Progression-Free Survival, TTP—Time to Progression, PR—Partial Response, SD—Stable Disease, RPA—Recursive Partitioning Analysis, DLT—Dose-Limiting Toxicity, TTFields—Tumor Treating Fields, BBB—Blood–Brain Barrier, IAEA—International Atomic Energy Agency, EPR—Enhanced Permeability and Retention.
